# Supplementary material for: Inflammatory factors and risk of lung adenocarcinoma: a Mendelian randomization study mediated by blood metabolites
Source: Front Endocrinol (Lausanne). 2024 Aug 27;15:1446863. doi: 10.3389/fendo.2024.1446863 (PMC11384989; doi:10.3389/fendo.2024.1446863)
Supplement: Supplementary file 1 [file DataSheet1.zip › supplementary files/S2/GCST90200256/forest.pdf]

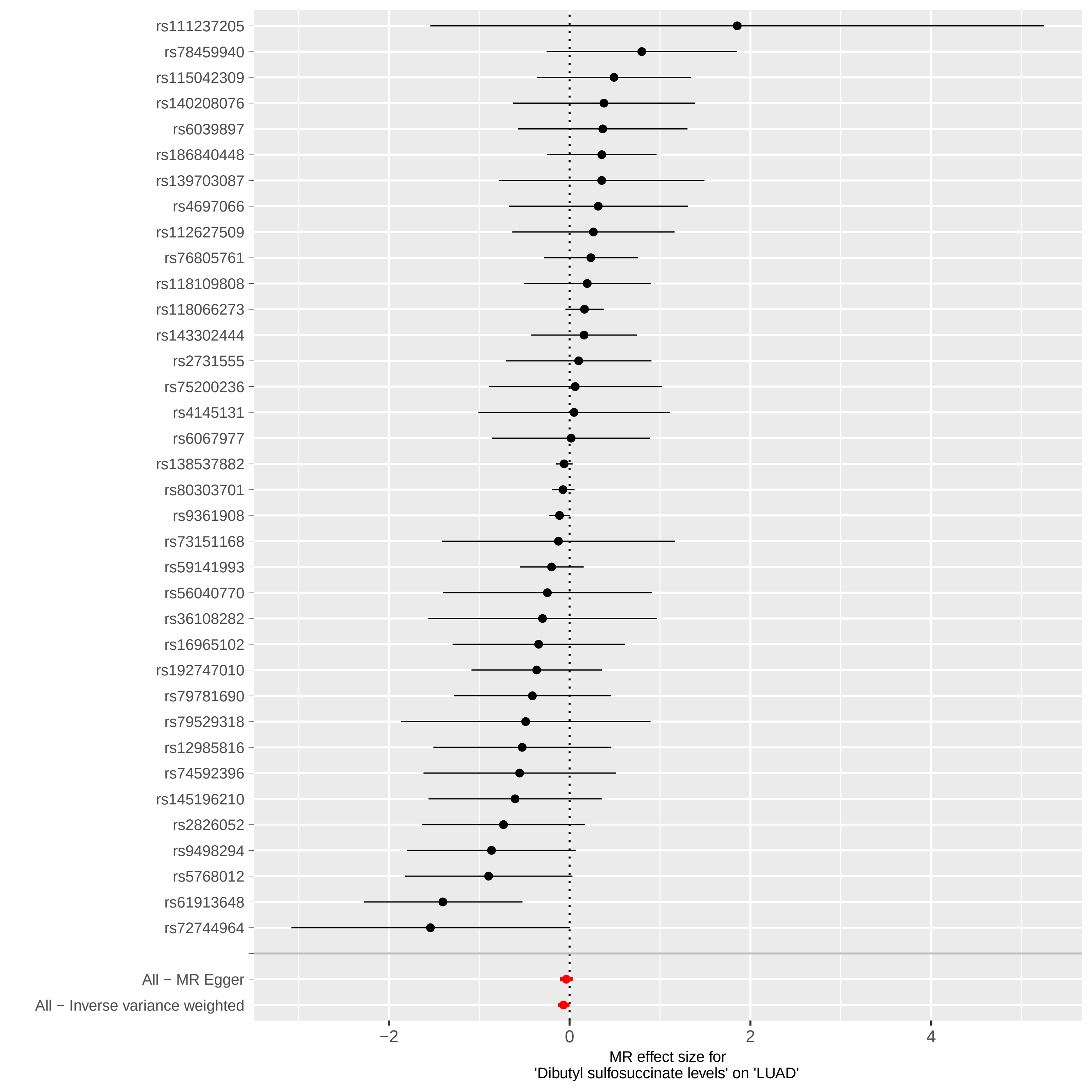
rs111237205
rs78459940
rs115042309
rs140208076
rs6039897
rs186840448
rs139703087
rs4697066
rs112627509
rs76805761
rs118109808
rs118066273
rs143302444
rs2731555
rs75200236
rs4145131
rs6067977
rs138537882
rs80303701
rs9361908
rs73151168
rs59141993
rs56040770
rs36108282
rs16965102
rs192747010
rs79781690
rs79529318
rs12985816
rs74592396
rs145196210
rs2826052
rs9498294
rs5768012
rs61913648
rs72744964
All − MR Egger
All − Inverse variance weighted
−2
0
2
4
MR effect size for
'Dibutyl sulfosuccinate levels' on 'LUAD'
